# Acute Mastitis

**Published:** 1892-01-09

**Authors:** 


### SURGERY.

### ACUTE MASTITIS.

It is not uncommon for boys at the age of puberty to suffer from induration and pain in the tissue under the nipple. This can often be traced either to a blow or to the friction of the braces against the front of the chest. Its predisposing cause, however, is the tendency which the mammary glands show at this age to abortive attempts at development. The complaint is sometimes troublesome, and requires an incision, but usually the application of a small boracic fomentation followed by a belladonna plaster is sufficient to effect a cure, the latter acting chiefly as a means of protection.

In women, the affection is fairly common during pregnancy, but occurs with the greatest frequency during lactation. Thus, Mr. John Birkett states that of 149 cases examined 132 occurred during suckling. This point is of interest as bearing upon its causation.

There are three varieties of acute mastitis usually given, (1) in the substance of the gland, (2) superficial to it, and (3) beneath it. M. Duplay in an interesting clinical lecture (reported in *Le Progres Medical* for November 14th 1891), points out that it is only the first of these, viz., the parenchymatous, that ought to be called mastitis. The other two are not in the gland at all, but commence in the cellular tissue round it. Superficial suppuration is often secondary to the more deeply-seated affection. Many causes have been assigned for the disease, such as cold, want of cleanliness, affections of the child's gums or saliva, and various injuries. The greatest importance, however, has been given to "cracks," or sore places on the nipple, which may act in two ways, first by giving a ready entrance to microbes, and secondly by necessitating an imperfect evacuation of milk, owing to the pain caused by the child's gums. This (according to Velpeau and others) induces a distension and finally a rupture of the galactopherous ducts with an escape of milk; and this causes the inflammation. This view has been widely adopted in this country, but in France many pathologists have advocated the view that the disease is essentially a lymphangitis, beginning by the absorption of the septic matter into these small fissures. M. Duplay, again, thinks that the symptoms are totally unlike those of a lymphangitis; at its outset it is much more insidious, and is not accompanied by rigors and enlargement of the axillary glands as we should expect in this disease. His theory is this: germs enter the ducts of the gland from the child's mouth or from other sources through the "cracks" in the skin of the nipple. These spread throughout the ramifications of the tubes even to the acini, giving rise to a true glandular inflammation.

We have, therefore, at least three distinct views as to the causation of acute parenchymatous mastitis: (1) It is a lymphangitis commencing by the absorption of septic matter from "fissures" on the nipple, &c. (2) It is a cellulitis due to the over-distension and rupture of milk ducts; and (3) the septic matter travels not by the lymphatics but in the tubes of the gland itself. Considering the method of onset, usually during lactation, the absence of glandular affection in the axilla, its somewhat slow increase, the firm "glandular" feeling of the breast, &c., we must rather incline to the last of these theories; and conviction is strengthened by two important facts. There are: (1) M. Budin has described a case where pus containing staphylococci could be readily squeezed from the ducts, and (2) Schlösser has found (as other observers have) both staphylococci and streptococci not only in the larger tubes, but even in the acini of the gland. To quote M. Duplay's words: "In the majority of cases microbes penetrate into the galactopherous ducts, with or without the aid of sore nipples; once there the inflammation may remain glandular or invade secondarily the cellular tissue." Of course "glandular inflammation" must mean a certain amount of involvement of connective tissue. If this be the pathology we can easily understand how ineffectual treatment must be in cutting short or curing the disease. Cold, warmth, belladonna, leeches, and many internal remedies have been tried with only poor success. There seem to be three methods, however, by which the affection may be modified. (1) *Evacuation* of the contents of the ducts by breast pumps. This should be done for a short period at frequent intervals. Over evacuation, by bringing on a renewed activity of the glandular elements, sometimes does more harm than good. This treatment may be supplemented by (2) *Methodical compression* by strapping and bandaging, together with careful "slinging up" of the breast. (3) *Cutting*. A few deep punctures may cause an exit for the *materies morbi*, and give speedy relief. It is not necessary to make these incisions in a radiating manner, for cutting across a duct or two will do more good than harm. In the latter stages, when fluctuation is present, the knife should certainly be used. As to the rival merits of poultices and cold (applied by icebags, &c.), it may be stated that neither appears to directly influence the course of the disease, unless it be an icebag kept constantly on in the initial stage; this is painful in most cases and is of doubtful efficacy. Poultices certainly relieve the pain, especially if they are very large and medicated with powdered hemlock leaves, or extract of belladonna and glycerine. But apart from this relief of pain they do no good apparently. Tonics and sedatives are often needed.

The other forms of acute mastitis are either secondary to the above or are due to septic infection of the lymphatics, &c., from excoriations or fissures. It should be remembered that there are numerous sebaceous glands in the nipple areola, and these may become blocked and inflamed, giving rise to a considerable amount of cellulitis. The treatment of superficial and deep "mastitis" (so-called), is that of ordinary cellulitis, incisions and drainage being urgently demanded when pus forms.

